# Beyond Aspiration Pneumonia: A 5-Year Diagnostic Delay in M. chimaera Lung Disease

**DOI:** 10.51894/001c.166009

**Published:** 2026-07-31

**Authors:** Nuran Sen, Kamal Khalil

**Affiliations:** 1 DMC - Sinai Grace

## 120

Mycobacterium chimaera is a slow-growing nontuberculous mycobacterium within the Mycobacterium avium complex (MAC), recognized as a distinct species in 2004. Although it became widely known after heater–cooler–associated cardiothoracic surgery outbreaks, it can also cause pulmonary disease in patients without prior cardiac procedures. Diagnosis is often delayed because cultures require several weeks to grow, and routine laboratory methods may not reliably distinguish M. chimaera from other MAC organisms.

A 50-year-old man with severe COPD on 3 L/min home oxygen and chronic dysphagia from childhood caustic ingestion, complicated by recurrent aspiration pneumonia, presented with five months of worsening productive cough, chills, night sweats without fever, and intermittent hemoptysis. CT chest revealed multiple right upper lobe cavitary lesions. Three initial AFB smears were negative, and he was treated for aspiration pneumonia and discharged on amoxicillin–clavulanate. One month later, final AFB culture from one of three sputum samples grew M. chimaera–intracellulare complex, but follow-up was unclear.

Four years later, he re-presented with worsening dyspnea and increased oxygen requirement. Imaging showed persistent right upper lobe cavitary disease with slight enlargement. Repeat cultures again grew M. chimaera, but treatment was declined. One year later, he was admitted with recurrent hemoptysis. Culture was negative; however, CT demonstrated stable cavitary bronchiectatic changes. Given repeated prior isolation and persistent cavitary disease, triple therapy with rifampin, ethambutol, and azithromycin was initiated for a planned 18-month course.

Diagnosis of NTM lung disease requires compatible symptoms, characteristic imaging (nodules, bronchiectasis, or cavities), and microbiologic confirmation. Typically, at least two positive sputum cultures are required. A single positive culture warrants further evaluation rather than dismissal. Cavitary MAC disease is considered a more aggressive phenotype, and treatment is generally recommended. Standard therapy for macrolide-susceptible MAC includes a macrolide, rifampin, and ethambutol for at least 12 months after culture conversion.

Negative AFB smears do not exclude NTM disease. Repeated isolation of M. chimaera with persistent cavitary abnormalities should prompt guideline-directed therapy and careful follow-up to avoid prolonged diagnostic delay.

